# What connectomics can learn from genomics

**DOI:** 10.1371/journal.pgen.1009692

**Published:** 2021-07-16

**Authors:** Patrick B. Chen, Jonathan Flint

**Affiliations:** Center for Neurobehavioral Genetics, University of California Los Angeles, Los Angeles, California, United States of America; The University of North Carolina at Chapel Hill, UNITED STATES

Geneticists, or perhaps more accurately genomicists, are used to big science, enormous projects that take years to complete and consume vast sums of money: sequencing mammalian genomes, genotyping a million people, obtaining ever more extensive catalogs of epigenetic marks, and so on. Last year the neuroscience community proposed something to rival the ambition of the human genome project. Entitled ‘The mind of a mouse’, a position paper in Cell described a visionary project to construct the map of the mouse brain down to the level of the synapse, requiring electron microscopy to obtain the necessary resolution [1]. With a price tag estimated at hundreds of millions of dollars, the amount of data to be generated makes the genome project look like the work of a miniaturist. “Roughly 1 million terabytes of data will need to be acquired”, and of course the project is only the beginning: unlike the identical connections that make up every worm brain (to date the only organism to have its connectome published), each mouse brain is unique, so “later work using the same brain mapping infrastructure will reveal aspects of neural circuits that are preserved from one animal to another, presumably based on inheritance, and importantly the ways in which connections vary between individuals, presumably based in part on different experiences”

There was a time when the generation of what is sometimes euphemistically called genome resource generation projects, including large-scale genome-wide association studies of disease, were decried as ‘fishing trips’ and contrasted with supposedly more impactful hypothesis-driven research. For those who recall those criticisms, it’s reassuring to discover that neuroscientists are now won over to the cause of resource generation. But it also raises an issue much discussed at the initiation of the Human Genome Project: what is industrial-scale science good for? And an ancillary, but equally important, question: is it worth the money?

There are many parallels between the Mind of a Mouse and the Human Genome Project: proof of principle experiments carried out in model organisms, the development of new and the improvement of old technologies, the realization that producing such vast amounts of data was going to place computational needs center stage, the promise of “discoveries … largely unexplainable in a previous era of investigation” [1] and community buy-in to protect the project from those who think the money would be better spent on other things. In the spirit of wanting to get the best science done, perhaps the genomics community can offer some advice to the connectomics community on how to do big science.

The first piece of advice has to do with the way a genomics resource is used. The genome project democratized genetic mapping and cloning. In the words of Francis Collins, the work of identifying mutations went from the perditional to the traditional [2]. Long ago, before we had physical maps, let alone genome sequence, the identification of a disease-causing mutation was the sort of success that would guarantee you a chair at a leading university. Nature, Cell and Science would be clamoring to publish your discovery. Now, with the genomes to hand, it’s a trivial job for a single laboratory. Mind you, this isn’t just because of the databases of assembled genomes that anyone with a computer terminal can access. It’s also due to the technological developments that the genomics projects supported, the development of automated sequencing machines and computer infrastructure that allows a single laboratory on its own to sequence entire genomes.

It would be great if connectomics could democratize neuroscience in the same way, but from the results from the pilot projects it’s hard to imagine that it will do so. For example, we now have a connectome of one half of the fly brain (about 22,500 neurons [3]) and of the mushroom body [4], the major site of associative learning in insects. This means that, if I were a fly researcher I would be in a position to identify connections for large areas of the brain, but once I had that circuit information, what will my next experiment be? The value of the electron microscopy data is the power it provides to understand circuitry, but it’s also essential to manipulate, to test hypotheses about how brains work. The genetics toolkit available in the fly, and worm, allows the manipulation of neurons and makes interventionist science possible. It’s almost inevitable that I’d want to create the connectome for a mutant, for a fly that has been subject to some experimental paradigm. And to do so, I’ll need to replicate all that expensive machinery and infrastructure belonging to the connectomics laboratories. That’s going to be even harder to do with the mammalian connectome. Of course, it’s possible that someone works out a quicker, cheaper, way to build a connectome so that the science does democratize. Perhaps tackling the connectome of the mouse will be an aspirational goal, focused on improving technology beyond the imaginable.

The second piece of advice follows from the first: that the generation of the resource is not divorced from the science to which it will ultimately be used. There’s no doubt that having a connectome will change neuroscience, but it’s not necessary to finish the whole mammalian connection then ask questions. It’s been difficult to use the connectome in the fly, and we have little clue how a connectome of the mammalian cortex is going to guide science. The fly projects demonstrate the value, the necessity, of asking questions as the project proceeds.

Geneticists routinely use the results of mega-science, the genome projects in their various incarnations, as tools to perform problem-centered science. There is no reason why a mammalian connectome project shouldn’t similarly bring together scientists working at different levels using cutting-edge technologies to solve a problem related to a mouse brain region, circuit or behavior. The real tragedy would be if the mouse connectome is created in a bubble, without connection to the genomics, and functional investigations that would make the breakthroughs we’d expect of a project on this scale. Rather than a detailed connectome of one C57BL/6J mouse brain, we need an organic mix of focused problem-based experimentation with high quality structured science approaches to provide a model for moving neuroscience forward in the 21st century: connectomics, circuit function, genetics, and molecular biology of a brain region or a behavior, tied closely together to address problems at multiple levels.

**Fig 1:**
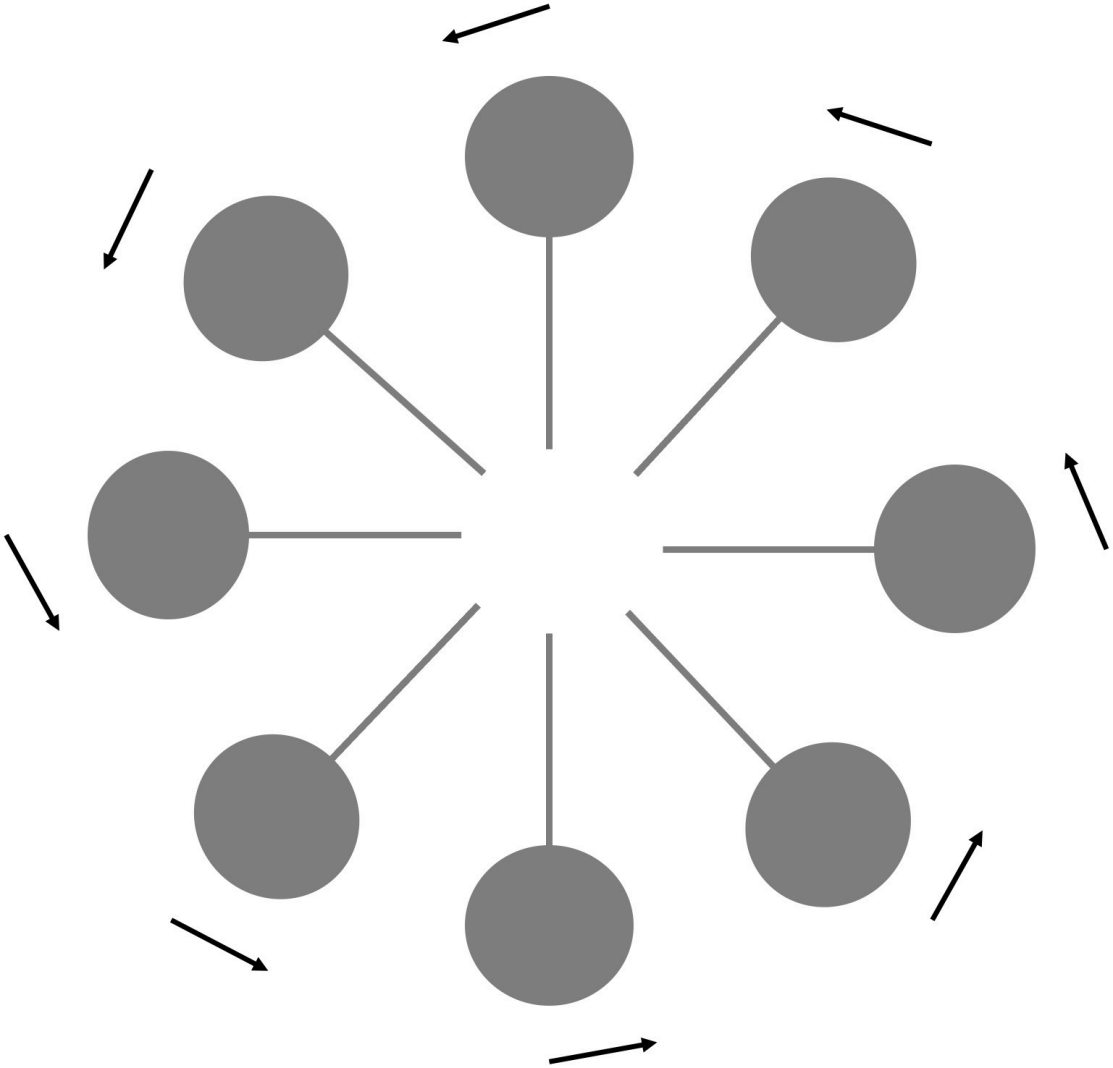
EP-G neurons in the fly ellipsoid body. Circles represent the cell bodies and bars represent dendrites, arranged in a ring. Neuronal activity in this ring, indicated by the black arrows, represents the direction of a fly’s movement.

There are instructive examples where connectomics has already moved the field forward. In the fly visual system there are separate pathways for recognizing on- and off-edge motion signals. The on-edge signals are locally computed on the dendritic arbours of columnar cells, called T4 cells. T4 cells respond to visual motion in a directionally-selective manner and have four subtypes, T4a-T4d. Each subtype is tuned to one of four cardinal directions. How do T4 cells compute the direction of motion? Before the fly connectome the eight classes of neurons were known, but not the mechanism. A high-resolution map revealed that the orientation of inputs to T4 subtypes reflects the direction to which they respond, so that inputs from the front of the neuron (relative to the animal) carry information about motion in that direction, and so on [5]. Cell type specific information and connections made it possible to test hypotheses about how motion direction works. Even more strikingly, a connectome revealed how flies orient themselves in space. Dendrites of a population of neurons called E–PG neurons in the fly’s ellipsoid body are arranged in a ring (see Fig 1). There is one ‘bump’ of activity in this ring which represents the direction of a fly’s movement. In short, the E–PG neurons are compass neurons, arranged appropriately as a compass [6,7]. In addition to the orientation and visual system examples, single behavior studies, combined with connectomics, have led to the discovery of a mechanism for sleep in flies [8], organizational principles governing how fruit flies groom their bodies [9], the identification of the neuronal basis of a distance-evaluation system [10] and given insights into the biology of aggression [11].

The value of integrating connectomics with a single functional question is made clear when we contrast the findings from the investigation of how the visual system works, and how the fly finds its way around the world, with the unbiased screen for circuit function carried out by activating subsets of neurons with genetic drivers [12]. That screen used GAL4 driver lines to obtain control over activity in neuronal populations [13]. Transgenes that turn off neuronal function were inserted into vast numbers of randomly selected neuronal patterns, and the flies were subjected to a battery of automated high throughput behavioral tests (think of this as a genetic screen in which the individual units are circuits, rather than genes). More than two thousand populations of neurons were genetically targeted, and behavioral traits were recorded in almost half a million flies. This heroic experiment identified regions of the fly brain involved in sensory processing, locomotor control, courtship, aggression, and sleep. But no novel mechanism was observed, no structure-function relationship similar to the compass discovery emerged.

What connectomics, and the associated technologies of circuit tracing and activation, teach us about the organizational principles of insect brains might not, of course, be informative of their performance in mammalian systems. Mammalian brains are likely not just more complex, they may have different design principles than insect brains. One example is their greater flexibility and redundancy. It’s perhaps not widely enough recognized that in the face of profoundly atypical neuroanatomy it’s possible to retain remarkably intact intellectual ability. Rare cases of hydrocephalus exist that result in massive enlargement of the ventricles and a brain with a very thin cortex [14], yet functionally appear relatively normal. Brain lesion studies teach the same lesson, of functional capacity maintained despite tissue loss [15,16].

At the end of a long description of attempts to reconstruct a piece of mouse neocortex at nanometer resolution [17], the authors posed the following question: “given the many challenges we encountered and those that remain in doing saturated connectomics, we think it is fair to question whether the results justify the effort expended. What, after all, have we gained from all this high-density reconstruction of such a small volume?” They answer their own question as follows: “we think that this “omics” effort lays bare the magnitude of the problem confronting neuroscientists who seek to understand the brain.” Hardly a ringing endorsement of the connectomics creed. And the densest mammalian connectome yet published (half a million cubic micrometers of cortical tissue yielding 2.7 m of neuronal cables and 153,171 synapses) [18] hasn’t led to major insights into how the brain works.

This brings us to the last piece of advice: involve geneticists. At some point the mammalian connectome will become a genomics project. It’s worth remembering that the human genome project went hand in hand with projects to create a compendium of sequence variants (the HapMap [19] and thousand genome’s projects [20]). The single biggest factor that determines variation in connectomes is heredity, something that is relatively easy to exploit in mice because of access to genetically inbred strains. There are 2,831 strains listed on the website of the Jackson laboratory (http://www.informatics.jax.org) and these are only a fraction of the total, if we include their derivatives, such as the recombinant inbreds (more than 100 BXD lines, mice descended from crosses between B6 and DBA/2J). The strains come from two species (*Mus musculus* and *Musculus spretus*) and at least three subspecies (*Mus musculus musculus*, *Mus musculus castaneus*, *Mus musculus domesticus*) [21]. The large amount of genetic variation that the inbred strains capture contributes to a remarkable array of behavioral and morphometric differences.

Variability is not limited to a handful of regions: cortical [22,23], hippocampal [24], subcortical [25], and sensory neurons [26] all vary with genetic background. The effect of genetic variation can even be seen in the total volume of structures; lateral septum volume can be ~68% as large in certain BXD lines compared to others [27], while basolateral amygdala volume can vary up to ~66% [28]. There are likely to be even more differences in cellular composition attributable to genetic variation in hitherto unanalyzed brain regions.

Since genetics has a such a large effect on the composition of cell type and brain volume, strain differences can be used to explore the consequences of changing circuit cellular composition and structure. For example, visual contrast in the mammalian retina is enhanced by a process known as lateral inhibition, where feedback loops between different cell types (such as rod and cone cells synapsing onto horizontal cells) ultimately serve to enhance firing of on-center cells and silence off-center cells to generate a visual receptive field [29]. Horizontal cells are a key cell type governing lateral inhibition to establish a receptive field. Based on these findings, circuit models have been generated on the assumption that the physical inputs and outputs from each cell type to each other cell type are conserved, with the ratio between inputs and outputs important and sensitive to the function of the network [30]. Yet some mouse strains have nearly twice as many horizontal cells or retinal ganglion cells than others; strains with high numbers of cells of one type do not necessarily also have high numbers of the other [31,32]. All of these animals have a functioning visual system, so how does the operation of the visual circuit change, given twice as many horizontal cells or retinal ganglion cells?

Similar questions about the relationship between circuit function and structure can be posed for behavior. For instance, low levels of activation of Vgat+ neurons in the posterodorsal subdivision of the medial amygdala lead to parental behaviors, while high levels lead to infanticide [33]. These switches from one behavior to another are believed to be due to changes in the number of activated neurons in a circuit, and the levels of activation within each neuron. With connectomic and cellular composition data indicating the appropriate strain differences, it becomes possible to test these hypotheses about how circuit architecture relates to function and behavioral output.

Brains, like genomes, need to be mapped. Cartographic tools transformed genetics, just as they will for neuroscience. There’s no doubting the value of having a connectome of the mammalian brain, but the manner of its doing and the use it will be put to are still matters for discussion. Our advice is to do it in collaboration with scientists who want to know what a particular circuit does, who want to know the biological basis of a behavior. A lesson from the fly connectome is that deferred gratification isn’t necessary, and might even be counterproductive; breakthroughs will come even when components of the connectome are complete. When we have a visual cortex, an amygdala, at what point do we need to commit to doing the whole mouse? Simply looking at a whole connectome won’t immediately suggest mechanism, unless we look with questions in mind, as the identification of the fly’s internal compass demonstrates. But even when we have such questions, the connectome is just the start. It’s an open question whether the connectome should be thought of as a technology project, whose aim is to put connectomics technology within reach of a single laboratory. But however that question gets answered, the hard questions of what circuits do, and how they do it, are still to be addressed, which is where genetics comes in, providing the tools to dissect mechanism.
